# CFTR regulates brown adipocyte thermogenesis via the cAMP/PKA signaling pathway

**DOI:** 10.1016/j.jcf.2022.08.012

**Published:** 2022-09-08

**Authors:** Kyung-Mi Choi, Sung-Hee Cho, Jung Hak Kim, Ae-Rhee Lilian Kim, Xiangmudong Kong, John C. Yoon

**Affiliations:** a Division of Endocrinology, Department of Internal Medicine, University of California Davis School of Medicine, Davis, California 95616, USA; b Institute of Molecular Biology and Genetics, School of Biological Sciences, Seoul National University, Seoul 08826, South Korea; c Department of Surgical and Radiological Sciences, University of California Davis School of Veterinary Medicine, Davis, California 95616, USA

**Keywords:** CFTR, thermogenesis, brown adipocyte, cAMP-PKA pathway

## Abstract

**Background:**

Cystic fibrosis (CF) is characterized by reduced growth and lower body weight, which are multifactorial. CF mouse models lack key disease characteristics that predispose to a negative energy balance, such as pulmonary infections or exocrine pancreatic insufficiency, and yet they still exhibit a growth defect and an abnormally increased energy expenditure. Whether adipocyte thermogenesis contributes to the elevated resting energy expenditure in CF mice is unknown.

**Methods:**

We examined the expression of CFTR in thermogenic brown adipose tissue (BAT) and investigated a functional role for CFTR using BAT-specific CFTR null mice (CFTR^BATKO^).

**Results:**

The CFTR protein is expressed in mouse BAT at levels comparable to those in the lungs. BAT-specific inactivation of CFTR in mice increases whole-body energy expenditure associated with sympathetic stimulation by cold exposure. Weight gain on a high-fat diet is attenuated in these mice. However, CFTR-deficient brown adipocytes themselves have impaired, rather than enhanced, thermogenic responses. These cells feature decreased lipolysis and blunted activation of the cAMP/PKA signaling pathway in response to adrenergic stimulation. This suggests that compensatory heat production in other tissues likely accounts for the increased systemic energy expenditure seen in CFTR^BATKO^ mice.

**Conclusions:**

Our data reveal a new role for CFTR in the regulation of adipocyte thermogenesis.

## 1. Introduction

Cystic fibrosis (CF) is a recessive lethal disease caused by a mutation in cystic fibrosis transmembrane conductance regulator (*Cftr*) gene, which encodes an anion (primarily chloride ion) channel regulating ion movement across the cell membrane [1]. CF affects multiple organs including lungs, pancreas, intestines, and male reproductive tract and leads to pulmonary infections, exocrine and endocrine pancreatic insufficiency, and male infertility [2–4]. CF patients also commonly exhibit poor growth and a lower body mass index (BMI) than age-matched controls, and a low BMI is associated with decreased lung function and increased mortality [5,6]. Thus, it is the standard of care to set BMI targets and encourage CF patients to consume a high-calorie diet to help meet the targets.

The etiology of the lower body weight in CF is multifactorial and involves both reduced caloric intake and increased energy expenditure [7]. The reduced intake can be due to malabsorption from exocrine pancreatic insufficiency, poor appetite from illness, and altered taste from sinus disease [8–10]. The increased energy expenditure specifically involves an elevation in the resting energy expenditure (REE), which refers to the energy cost of physiological functions needed to maintain homeostasis and excludes the energy cost of voluntary physical activity. A number of studies have suggested that CF patients have increased REE, ranging from 4% to 33% higher compared to controls [11–14]. Oxygen consumption by respiratory muscles does not explain the increased REE [14].

Animal models of CF all show reduced body weight, including mouse, rat, pig, and ferret [15–19]. Mouse models of CF do not develop chronic pulmonary infections or exocrine pancreatic insufficiency [20,21], thus eliminating two major causes of a negative energy balance, but CF mice still exhibit low body weight and growth impairment. The F508del mice, which carry the mouse equivalent of the most studied human CFTR gene mutation, have a growth impairment that persists even when functional CFTR expression is restored in the gut by transgene expression in the intestinal epithelium [22,23]. Reduced insulin-like growth factor-1 (IGF-1) levels have been implicated in the low body weight [22]. But the growth defect is already manifest in the perinatal period, when the IGF-1 levels are very low with little difference between the mutant and control animals, suggesting that other mechanisms contribute to the low body weight phenotype [22]. The F508del mice have also been shown to display increased energy expenditure [23].

Thermogenic adipocytes increase the REE via adaptive thermogenesis, which involves regulated heat production in response to environmental signals such as a drop in ambient temperature or nutritional excess [24,25]. Recent studies indicate that thermogenic adipocytes are much more extensive in the human body than previously realized and can significantly impact cardiometabolic health [26,27]. Thermogenic brown adipose tissue (BAT) develops through late gestation in humans and mice [28] and is more prevalent in younger individuals who make up most of the CF patient population. Based on our observation that the CFTR protein is expressed in BAT in mice, we decided to investigate the possibility that loss of CFTR function selectively in BAT alters systemic energy expenditure and body weight. We generated BAT-specific CFTR knockout mice (CFTR^BATKO^) and report here that CFTR^BATKO^ mice exhibit higher energy expenditure compared to their littermate controls and have an increased capacity for thermogenesis upon cold stimulation. CFTR^BATKO^ mice gain less weight than controls on a high-fat diet (HFD). Interestingly, we also find that primary brown adipocytes lacking CFTR function have reduced activation of the cAMP-PKA signaling pathway and decreased thermogenic respiration, suggesting that thermogenesis in other tissues besides BAT may underlie the increase in systemic energy expenditure. Our observations point to a previously unappreciated involvement of CFTR in regulating adipocyte thermogenesis and have implications for developing therapeutic approaches to manipulate energy expenditure in CF.

## 2. Materials and methods

### 2.1. Animals

Animal experiments were performed according to the procedures approved by the University of California Davis Institutional Animal Care and Use Committee (IACUC). The floxed CFTR mice (Cftr ^*tm1Cwr*^) were obtained from Case Western Reserve University [16]. The Ucp1-Cre mice were acquired from the Jackson Laboratory (stock 024670). All mice used in this study were on the C57BL/6J background. Unless otherwise noted, mice were maintained on standard chow (Teklad Global Rodent Diets) at 22 °C on a 12 h light/dark cycle. For a high-fat diet (HFD), mice were fed a diet with 60% fat (Research Diets, D12492). All experiments were performed with age-matched BAT-specific CFTR KO (CFTR^BATKO^) and wild-type (WT) littermate mice.

### 2.2. Isolation and differentiation of brown preadipocytes

Stromal vascular fractions (SVF) from BAT of 4-week-old male CFTR^BATKO^ or WT littermate mice were prepared according to published protocols [29]. Briefly, freshly isolated adipose tissues were minced and digested in a collagenase D/dispase II-containing digestion buffer. SVF was collected by centrifugation and plated in 100 mm plates. At approximately 80% confluency, cells were treated with 0.25% trypsin-EDTA and replated at 500,000 cells/well into 6-well plates. Differentiation was induced (day 0) by treating fully confluent cells with induction medium (Dulbecco’s Modified Eagle Medium (DMEM) containing 10% fetal bovine serum (FBS), 5 μg/ml insulin, 0.5 mM isobutylmethylxanthine (IBMX), 5 μM dexamethasone, 125 μM indomethacin, 1 nM 3,3’,5-triiodo-L-thyronine (T3) and 1 μM rosiglitazone) for 2 days and cells were then maintained in a medium containing 5 μg/ml insulin, 1 nM T3 and 1 μM rosiglitazone until analysis.

### 2.3. Body composition and indirect calorimetric analysis

Body composition was measured by dual-energy X-ray absorptiometry (DEXA) under isoflurane anesthesia, using a Lunar PIX-Imus II Densitometer (GE Medical Systems).

Energy expenditure, food intake, and physical activity were evaluated in CFTR^BATKO^ and WT-littermate mice by indirect respiratory calorimetry in the Comprehensive Lab Animal Monitoring System (CLAMS, Columbus Instruments). Animals were acclimated to the facility for 1 week prior to initiation of calorimetry at 13 weeks of age. Calorimetry data were collected for 48 h at three environmental temperatures (30, 22, and 10 °C).

### 2.4. Acute cold exposure and body temperature measurement

CFTR^BATKO^ and WT male mice at 10 weeks were singly housed in cages at 10 °C without food and with free access to water. The core rectal temperature was measured every hour using a thermometer (Bioseb).

### 2.5. Tissue temperature recording

Mice were anesthetized with 1–4% isoflurane and a thermocouple probe (Sable Systems International) was implanted in BAT. Real-time changes in BAT temperature following intraperitoneal injection of CL316,243 (1 mg/kg body weight) were recorded with a TC-20 0 0 thermocouple meter (Sable Systems International).

### 2.6. Statistics

Data are presented as mean ± s.e.m. For a single comparison, we performed the two-tailed Student’s *t*-test. Two-way ANOVA analysis was performed using GraphPad Prism version 8.2 (GraphPad Software) for energy expenditure, body weight, glucose tolerance, and tissue temperature data.

## 3. Results

### 3.1. Brown fat-specific ablation of CFTR increases systemic energy expenditure and adaptive thermogenesis

**Fig. 1.**
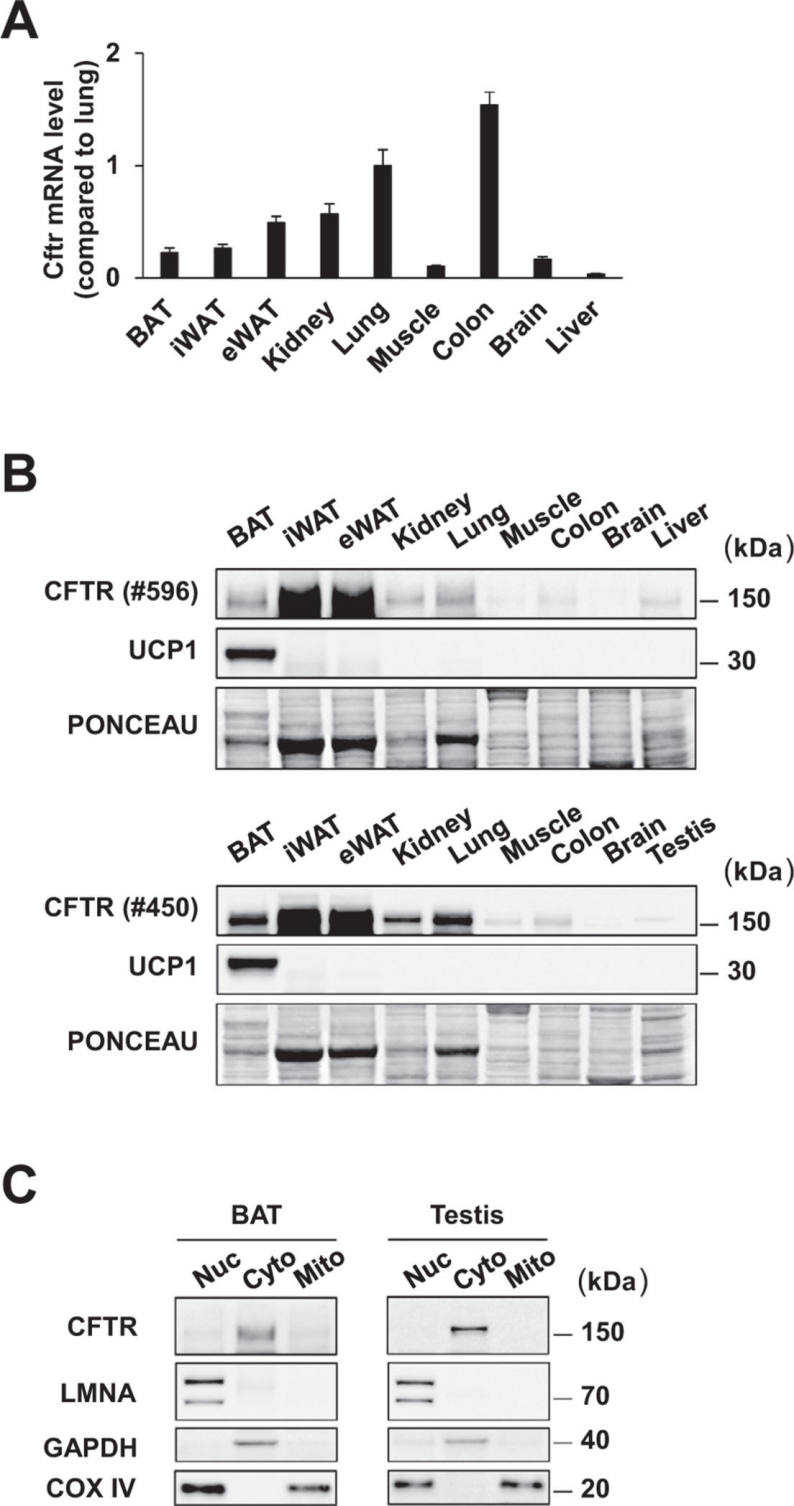
CFTR is expressed in thermogenic brown fat in mice. (A) mRNA expression of CFTR gene in mouse tissues detected by qPCR using a primer pair targeting the 3’-UTR region of the CFTR gene. n = 5. Data are presented as mean ± s.e.m. (B) Immunoblotting of CFTR protein in mouse tissues with the CFTR monoclonal antibodies #596 and #450. Ponceau S staining is used as a loading control. (C) Immunoblotting of nuclear (Nuc), cytosolic/microsomal (C/M), and mitochondrial (Mito) fractions for CFTR protein with the CFTR monoclonal antibody #450. LMNA, nuclear marker; GAPDH, cytosolic marker; COX IV, nuclear/mitochondrial marker.

Because adipocyte thermogenesis is an important component of the systemic energy expenditure in mice at room temperature (22 °C), we considered the possibility that loss of CFTR activity in BAT may impact adipocyte thermogenesis and thereby contribute to the elevated energy expenditure in CF mice. The CFTR mRNA was detected in all mouse adipose depots examined, including BAT, inguinal white adipose tissue (iWAT) and epididymal white adipose tissue (eWAT) (Fig. 1 A). We also assessed protein expression using two different CFTR monoclonal antibodies directed at the regulatory domain and the second nucleotidebinding domain of the CFTR protein, and both antibodies detected CFTR in mouse BAT at levels comparable to those in the lungs (Fig. 1 B). Subcellular fractionation with mouse adipose tissues showed that the CFTR protein localizes to the cytosolic/microsomal fraction in BAT, as expected (Fig. 1 C). We decided to investigate the functional consequences of selectively inactivating CFTR in brown adipocytes.

**Fig. 2.**
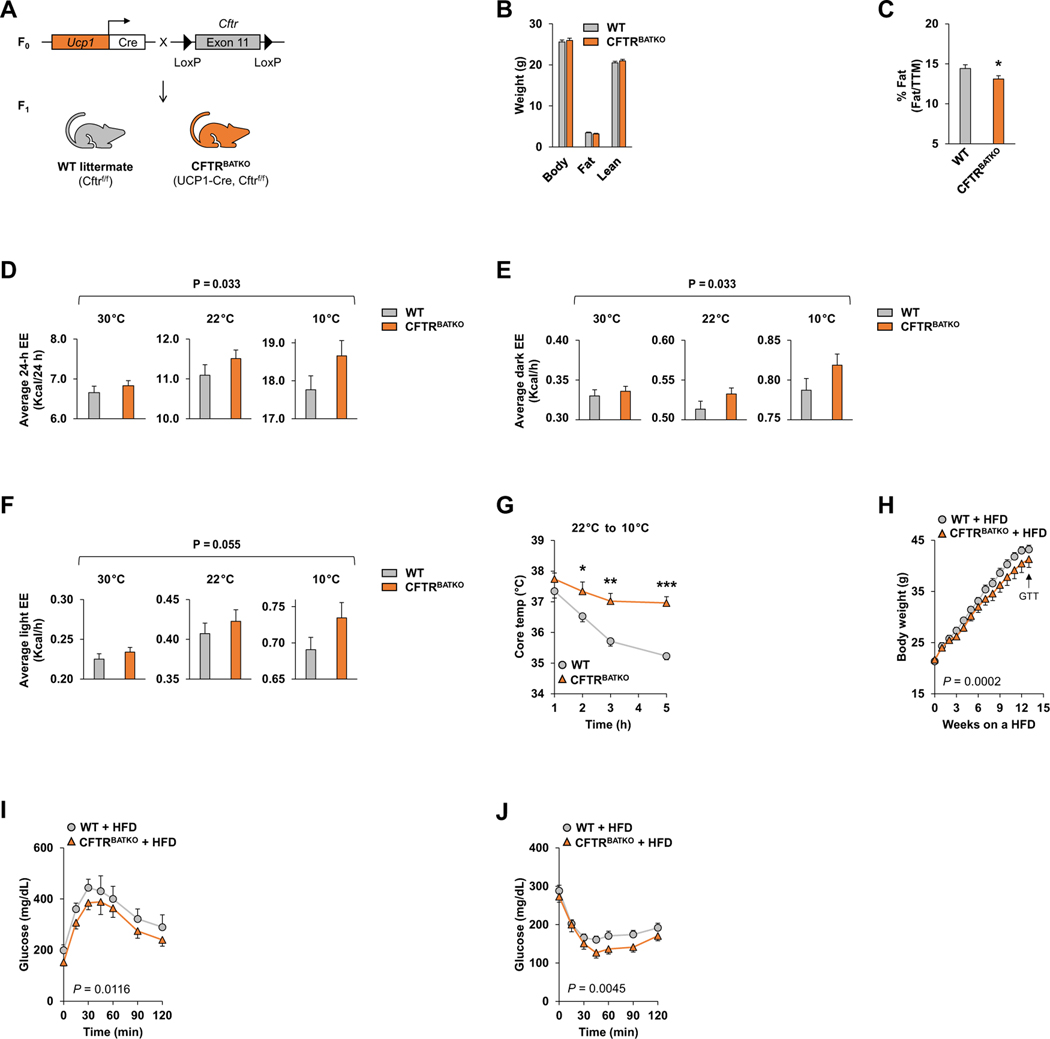
Genetic disruption of CFTR in BAT enhances whole-body thermogenesis and attenuates diet-induced obesity. (A) Breeding strategy to generate CFTR^BATKO^ mice (Ucp1-Cre, CFTR ^f/f^) and their WT littermates (CFTR ^f/f^). (B) Body composition analysis by DEXA in 11-week-old male CFTR^BATKO^ mice and WT littermate controls fed regular chow at 22 °C. n = 9 per group. (C) Percent body fat normalized to total tissue mass (TTM). n = 9 per group. (D) Average 24-hour energy expenditure (Kcal/24 h), (E) average dark cycle energy expenditure (Kcal/h), and (F) average light cycle energy expenditure (Kcal/h) in 11-week-old male CFTR^BATKO^ mice and WT littermate controls at 30 °C, 22 °C, and 10 °C environmental temperatures; n = 9 per group. ANOVA *p* -value is determined by a comparison between WT and KO across 30 °C, 22 °C, and 10 °C. (G) Core rectal temperature of singly housed mice upon transition from 22 °C to 10 °C. WT, n = 7; CFTR^BATKO^, n = 5. (H) Body weight of CFTR^BATKO^ and WT mice during HFD feeding. WT, n = 9; CFTR^BATKO^, n = 10. The black arrows indicate the time when the glucose tolerance test (GTT) and insulin tolerance test (ITT) were performed. (I) GTT in CFTR^BATKO^ and WT mice after 13 weeks of HFD. WT, n = 6; CFTR^BATKO^, n = 8. (J) ITT in CFTR^BATKO^ and WT mice after 14 weeks of HFD. WT, n = 8; CFTR^BATKO^, n = 10. Data are presented as mean ± s.e.m. *P* -values are determined by two-tailed Student’s t-test (B, C, G), two-way ANOVA with post-hoc Tukey test (D, E, F) or two-way ANOVA followed by Fisher’s LSD test (H, I, J). *p < 0.05; **p < 0.01; ***p < 0.001.

We generated a BAT-specific CFTR null mouse model (UCP1-Cre; CFTR ^f/f^ or CFTR^BATKO^) by breeding the floxed Cftr mice (Cftr ^*tm1Cwr*^), which have exon 11 (exon 10 in legacy nomenclature numbering) floxed, with the Ucp1-Cre mice (Fig. 2 A). Cre-mediated removal of this floxed allele results in an in-frame deletion and loss of CFTR function [16] and has been used by other investigators to inactivate CFTR in a tissue-specific fashion [31–33]. Because uncoupling protein 1 (Ucp1) is primarily expressed in brown adipocytes, the exon 11-deleted allele is present in BAT but not in iWAT or eWAT (Supplementary Fig. 1). Lower levels of the UCP1 mRNA have also been detected in some other tissues such as adrenal gland and mammary gland, but these tissues express little CFTR [34,35]. We confirmed the expression of the Cre recombinase and the deletion of CFTR in BAT from CFTR^BATKO^ mice (Supplementary Fig. 2A, B). Whereas global CFTR null mouse models typically develop intestinal obstruction and premature death shortly after weaning [14,30], CFTR^BATKO^ mice did not exhibit a significant difference from their wild-type (WT) littermates (CFTR ^f/f^) in viability or body weight on a regular diet (Fig. 2 B). The percentage of fat mass relative to the total tissue mass on DEXA was modestly lower in CFTR^BATKO^ mice (Fig. 2 C).

When analyzed by indirect calorimetry, CFTR^BATKO^ mice had a higher average 24-hour energy expenditure across temperatures compared to WT littermates (Fig. 2 D), as well as a higher dark cycle energy expenditure (Fig. 2 E) and a strong trend toward a higher light cycle energy expenditure (Fig. 2 F). The difference in energy expenditure between WT and CFTR^BATKO^ mice appeared to get larger at lower temperatures, suggesting that loss of CFTR becomes functionally significant under conditions of elevated adrenergic signaling. Oxygen consumption and carbon dioxide production showed a similar pattern without reaching significance (Supplementary Fig. 3A, B). No differences were seen in the respiratory exchange ratio (RER), food intake, physical activity, or carbohydrate and fat oxidation rates between WT and CFTR^BATKO^ (Supplementary Fig. 3C–G). Because of the apparent dependence on adrenergic stimulation, we subjected CFTR^BATKO^ mice and their littermates to cold stress, which strongly activates sympathetic output to BAT. Upon acute cold exposure at 10 °C, CFTR^BATKO^ mice were better able to maintain their core body temperature than WT mice, which dropped their core temperature by about 2 °C over several hours (Fig. 2 G). The expression of the UCP1-Cre transgene by itself did not affect systemic energy balance or cold tolerance in mice (Supplementary Fig. 4A–F). These data indicate that deleting the CFTR gene in BAT increases whole-body energy expenditure and enhances heat production.

In addition to mounting a thermogenic response to temperature changes, BAT is also viewed as playing a role in diet-induced thermogenesis [36, 37]. On a high-fat diet (60% fat), CFTR^BATKO^ mice gained less weight than WT control littermates (Fig. 2 H) despite having the same amount of food intake (Supplementary Fig. 3H). Furthermore, CFTR^BATKO^ mice displayed enhanced glucose tolerance and insulin sensitivity compared to WT littermates (Fig. 2 I,J), which are likely a consequence of the lower body weight. These data uncover a new role for BAT CFTR in regulating whole-body energy metabolism.

### 3.2. CFTR deficiency impairs thermogenesis in BAT and isolated primary brown adipocytes

**Fig. 3.**
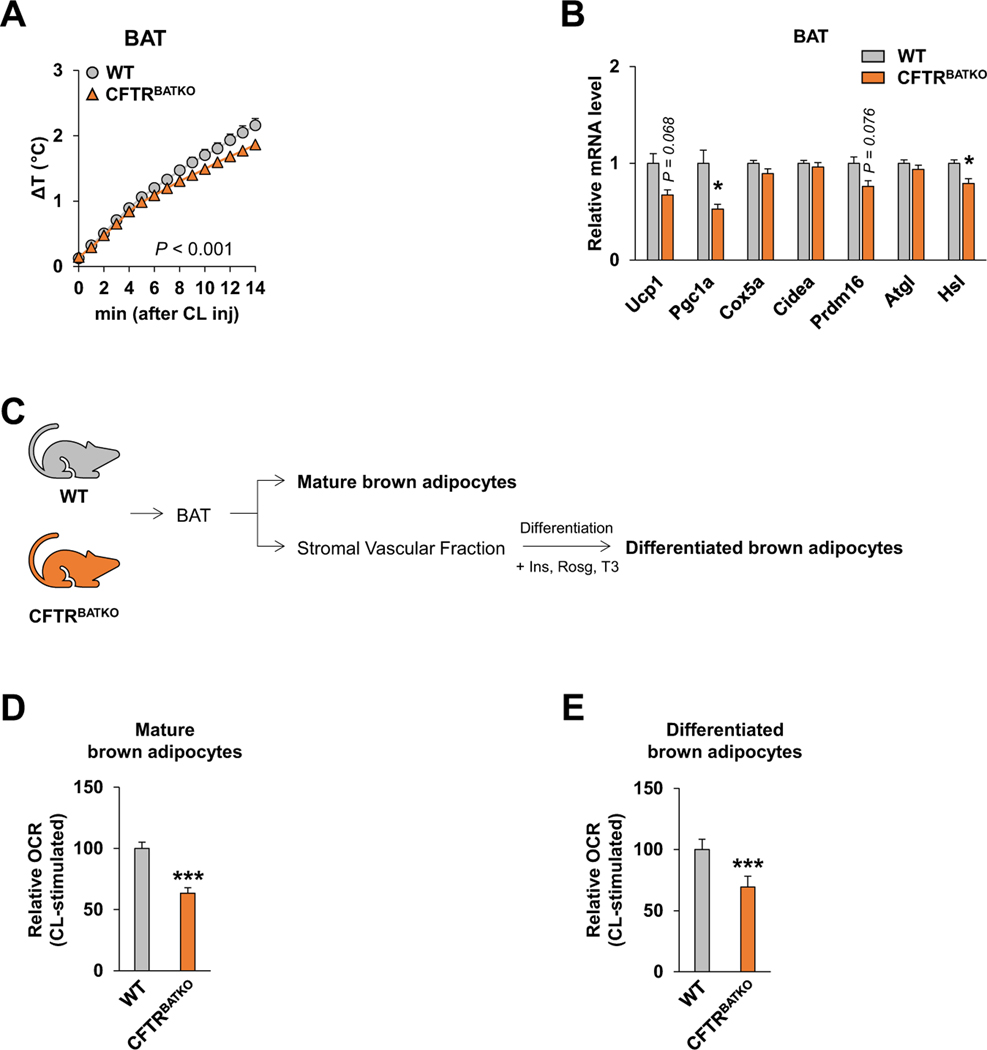
BAT and brown adipocytes from CFTR^BATKO^ mice exhibit impaired thermogenic response to adrenergic stimulation. (A) Changes in BAT temperature following intraperitoneal CL316,243 injection (1 mg/kg) in CFTR^BATKO^ and WT littermate controls. n = 4 for both groups. (B) Real-time qPCR analysis of thermogenesis- and lipolysis-related genes in BAT from 10-week-old male CFTR^BATKO^ (n = 7) and WT-littermates (n = 8) after 5 hours of acute cold exposure at 10 °C. (C) Isolation of mature brown adipocytes and the stromal vascular fraction (SVF) from BAT for measurements of the oxygen consumption rate (OCR). Ins, Insulin; Rosg, Rosiglitazone; T3, Triiodothyronine. (D) OCR measurements in mature brown adipocytes isolated from CFTR^BATKO^ or WT mice. Cells were treated with 10 μM CL316,243 (CL) and immediately used for OCR measurements. n = 4 for both groups. (E) OCR measurements in fully differentiated brown adipocytes derived from CFTR^BATKO^ BAT compared to WT BAT. CL316,243 (10 μM) was used for *β*3-adrenergic stimulation. n > 3 for each group. Data are presented as mean ± s.e.m. *P* -values are determined by two-tailed Student’s t-test. *p < 0.05; ***p < 0.001.

To assess local tissue heat generation in response to adrenergic activation, we implanted a thermocouple probe in BAT and monitored real-time changes in BAT temperature following intraperitoneal administration of the *β*3-adrenergic agonist CL316,243. Unexpectedly, the rate of temperature increase in BAT was lower in CFTR^BATKO^ mice (Fig. 3 A). We also examined the BAT expression levels of several adipocyte thermogenesis- and lipolysis-related genes following acute cold exposure and found PGC1 α and HSL were lower in CFTR^BATKO^ BAT compared to WT BAT, while others such as Ucp1 and Prdm16 showed a trend toward downregulation (Fig. 3 B). These observations suggest that the systemic effects of BAT CFTR ablation on thermogenesis may not be cell autonomous.

Next, we isolated mature adipocytes and stromal vascular fraction (SVF) from BAT in CFTR^BATKO^ mice and WT littermates (Fig. 3 C) to assess adrenergic receptor-mediated thermogenic respiration. We observed a significantly lower OCR in CFTR-deficient mature brown adipocytes relative to WT brown adipocytes (Fig. 3 D) following *β*3-adrenergic stimulation with CL316,243. Similarly, brown adipocytes differentiated in culture from CFTR^BATKO^ BAT SVF consistently showed a lower OCR relative to those differentiated from WT BAT SVF (Fig. 3 E). These results indicate that loss of CFTR in brown adipocytes reduces their thermogenic capacity.

### 3.3. Loss of CFTR dampens activation of the cAMP-PKA signaling pathway in brown adipocytes

**Fig. 4.**
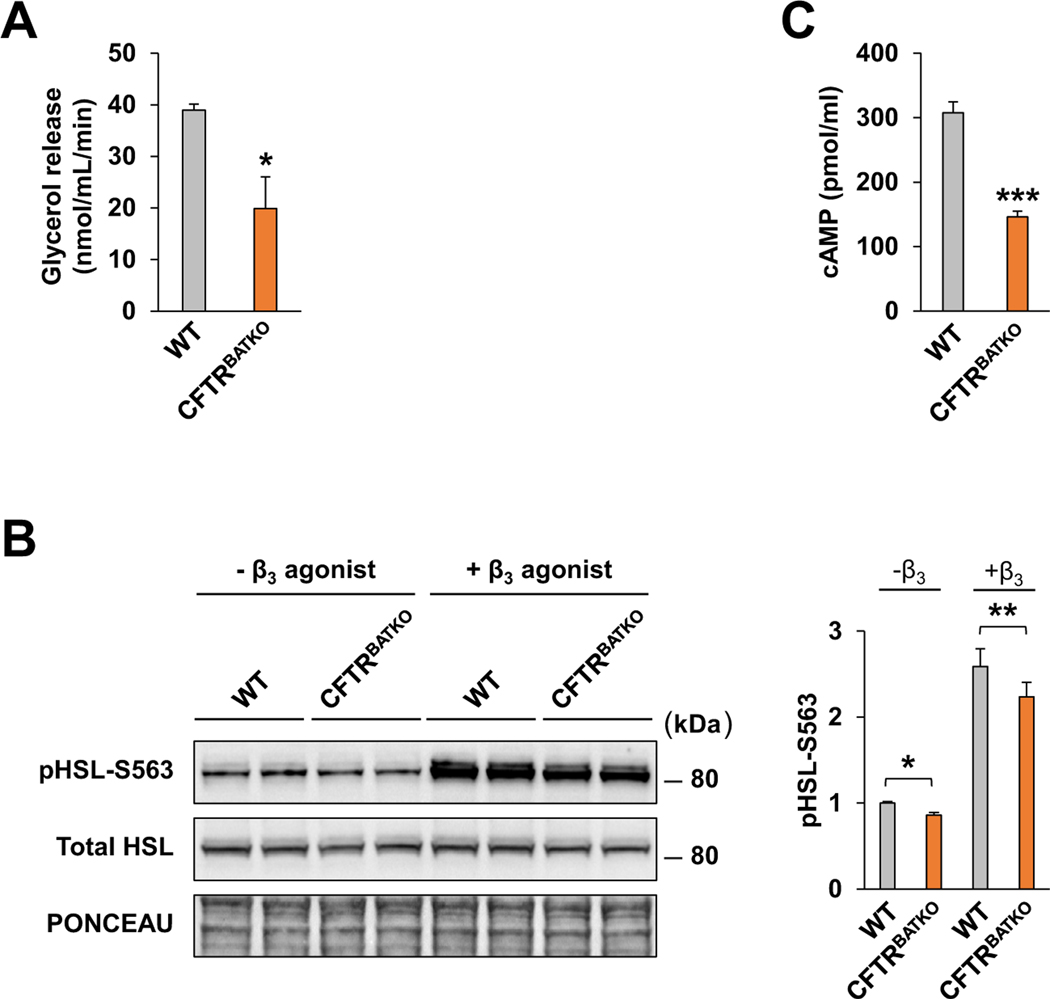
CFTR deficiency in brown adipocytes reduces activation of the canonical adrenergic signaling pathway. (A) Lipolysis rate of differentiated brown adipocytes derived from CFTR^BATKO^ and WT-littermates following 10 μM CL316,243 stimulation. WT, n = 4; KO, n = 3. (B) Immunoblot of total and phosphorylated HSL in differentiated brown adipocytes with or without CL316,243 treatment (10 *μ*M). Bar graph indicates quantification of phospho-HSL in the blot (n = 4 for each group). (C) cAMP levels of differentiated WT and CFTR^BATKO^ brown adipocytes treated with CL316,243 and IBMX (n = 4 for each group). Data are presented as mean ± s.e.m. *P* -values are determined by two-tailed Student’s t-test. *p < 0.05; **p < 0.01; ***p < 0.001.

*β*3-adrenergic signaling is considered to be a dominant pathway governing brown adipocyte thermogenesis in response to the environment [38]. Elevated levels of cAMP that result from *β*3-adrenergic stimulation promote activation of protein kinase A (PKA), which targets downstream signaling molecules such as p38 mitogen activated kinase (MAPK), cAMP response element binding protein (CREB), and hormone sensitive lipase (HSL), thus impacting multiple aspects of adaptive thermogenesis including lipolysis, mitochondrial regulation, and increased Ucp1 expression and activity. To understand why loss of CFTR reduces thermogenic respiration in brown adipocytes, we examined the lipolysis rate, HSL phosphorylation, and cAMP levels (Fig. 4 A,B,C). CFTR-deficient brown adipocytes exhibited a significantly decreased lipolytic response after *β*3-adrenergic stimulation (Fig. 4 A), as well as reduced HSL phosphorylation (Fig. 4 B) and lower cAMP levels (Fig. 4 C). These results are consistent with blunting of the cAMP-PKA signaling pathway and provides an explanation for the impairment of thermogenesis in CFTR null brown adipocytes.

## 4. Discussion

In this study, we have identified a new functional role for CFTR in brown adipocyte physiology and the regulation of whole-body energy balance using a mouse model. Because CF is a multisystem disease, the consequences of CFTR dysfunction have been investigated in many epithelial cell types ranging from the airways, intestines, pancreas, and reproductive tracts to sweat glands. However, its role in adipocytes has received relatively little attention. Bederman et al. [39] previously reported CFTR mRNA expression in visceral white fat in mice and observed that CF adipocytes were smaller in size than WT adipocytes due to lower triglyceride storage, while the number of adipocytes was not affected. Because this low triglyceride storage phenotype could be overcome under conditions of carbohydrate overfeeding, excess lipids, and lower energy expenditure, it was suggested that limited substrate availability in CF mice was a driving factor in the low adiposity [39,40]. Reducing systemic energy expenditure could therefore be key to restoring adiposity in CF mice. Our study indicated that CFTR is also expressed in BAT, a tissue that specializes in adaptive thermogenesis, raising the question of whether loss of CFTR affects BAT thermogenesis. Using conditional knockout mice, we have demonstrated here that inactivation of CFTR in BAT perturbs brown adipocyte thermogenic function as well as increasing systemic energy expenditure, which is evident in the setting of increased adrenergic signaling such as cold exposure. These observations highlight the physiological significance of CFTR function in BAT.

**Fig. 5.**
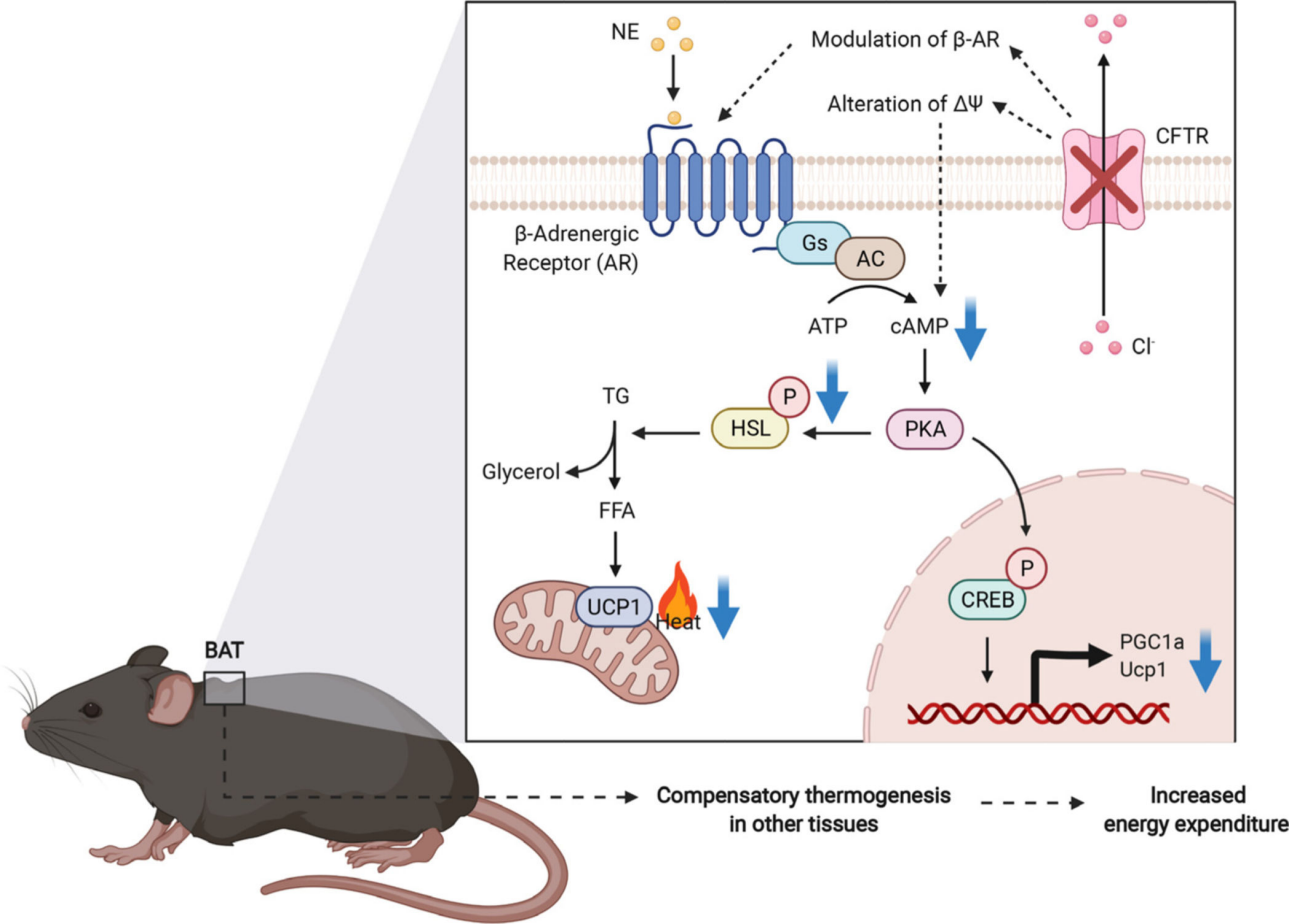
A schematic diagram of the impact of CFTR loss on the cAMP-PKA signaling pathway and systemic thermogenesis.

Under environmental conditions in which cold-induced thermogenesis is activated, it becomes clear that loss of CFTR in BAT increases the energy expenditure and predisposes mice to developing a negative energy balance. This initially suggested to us that CFTR may have an inhibitory role in brown adipocyte thermogenesis and CF mice have overactive BAT that dissipates excessive heat and wastes calories. Contrary to our expectations, however, primary brown adipocytes isolated from CFTR^BATKO^ mice displayed diminished thermogenic capacity as well as reduced activation of the cAMP-PKA pathway. Reconciling this with the increased energy expenditure at the systemic level entails other compensatory mechanisms of heat production (Fig. 5). With UCP1 knockout mice, which have profoundly impaired BAT thermogenesis, multiple compensatory thermogenic mechanisms have been proposed, including shivering thermogenesis in skeletal muscle, elevated proton leak in muscle mitochondria, and alterations in iWAT such as browning and UCP1-independent thermogenesis [41]. Because mice are at thermoneutrality at 30 °C, an ambient temperature of 22 °C requires active thermogenesis to maintain the core temperature, and a reliance on noncanonical mechanisms not mediated by UCP1 in BAT may lead to additional energy expenditure and heat dissipation. UCP1 null mice are resistant to diet-induced obesity regardless of the environmental temperature [42], suggesting that comparable mechanisms may be operational in the context of diet-induced thermogenesis. In a similar vein, impaired BAT thermogenesis in CFTR^BATKO^ mice may lower the metabolic efficiency and necessitate greater amounts of ingested energy per body mass gained. This can potentially explain the paradoxical increase in whole-body energy expenditure. Because UCP1 is found in beige adipocytes, loss of CFTR in these cells may also become significant, especially under cold temperature conditions, and result in remodeling of beige fat that contributes to greater energy expenditure.

Adrenergic signaling modulates CFTR activity via PKA- dependent activation of CFTR [43,44]. We have obtained evidence that loss of CFTR in BAT blunts adrenergic signaling, but it remains unclear how CFTR regulates the intracellular cAMP level in brown adipocytes. CFTR can interact with other membrane proteins and has been reported to exist in a macromolecular complex with the β2-adrenergic receptor in airway epithelial cells [45]. CFTR has also been shown to increase the surface density of the adenosine 2B receptor (A2BR) and enhance the adenosine-induced cAMP response [46]. Thus, it is conceivable that CFTR directly modulates the β3-adrenergic receptor in brown adipocytes. Another possibility is that CFTR may affect the membrane potential directly or indirectly, changing intracellular calcium influx and thereby cAMP production. For example, it has been reported that a potassium channel, KCNK3, blocks membrane depolarization in brown adipocytes [47], lessening calcium entry through voltage- dependent calcium channels and attenuating cAMP-PKA signaling. Electrophysiological studies of CFTR null brown adipocytes may shed light on these possibilities.

While CF mice do not faithfully reproduce all aspects of human CF, they exhibit increased energy expenditure and low weight gain. Both humans and mice possess constitutively active and inducible thermogenic fat [48,49], and recent data have highlighted the importance of human BAT in metabolic health [26]. In humans, the CFTR mRNA expression in deep neck BAT is higher than in subcutaneous WAT [50]. However, there still remain some differences between humans and mice. At room temperature, humans do not need to activate adipocyte thermogenesis to maintain the core temperature, unlike mice, so whether a thermogenic defect in brown adipocytes will trigger compensatory thermogenic mechanisms in other tissues is uncertain. BAT thermogenic defects are still relevant in the context of diet-induced thermogenesis and may help explain the lower weight gain on a calorie dense diet [51].

It would be of interest to know how CFTR modulator drugs affect CF BAT function and systemic energy expenditure. Recent studies in CF patients suggest that CFTR modulator drugs, which are likely to be the mainstay of CF treatment in the future, confer multiple extrapulmonary beneficial effects, including enhanced nutritional status [7]. One small clinical study documented a reduction in REE following ivacaftor treatment [52]. Because mouse CFTR does not respond well to the modulator drugs, it would be appropriate to utilize the humanized F508del mouse model which carries the human CFTR F508del transgene in a mouse CFTR null background and also exhibits growth retardation [53].

In conclusion, we have shown a novel role for CFTR in thermogenesis and adrenergic signaling in brown adipocytes. Given the emerging importance of human thermogenic fat in metabolic health, a better knowledge of how CFTR controls brown adipocyte physiology and systemic energy balance will be valuable for the development of new approaches to prevent and treat CF-associated metabolic complications.

## Supplementary Material

Supplementary Material
