# Supplementary material for: CFTR regulates brown adipocyte thermogenesis via the cAMP/PKA signaling pathway
Source: J Cyst Fibros. Author manuscript; Available in PMC 2023 Jul 10. (PMC10332805; doi:10.1016/j.jcf.2022.08.012)
Supplement: Supplementary Material [file NIHMS1903120-supplement-Supplementary_Material.docx]

**SUPPLEMENTARY MATERIAL**

**Supplementary Methods**

**Preparation of mature brown adipocytes**

BAT was collected, minced, and digested as described in the isolation of SVF. After centrifugation, mature adipocytes in the upper layer were collected and washed with culture media (DMEM with 10% FBS). A syringe was used for the removal of the infranatant. Mature adipocytes were then suspended in culture media for analysis.

**Quantitative PCR**

Total RNA was extracted from tissues using TRIzol reagent (Invitrogen). For qPCR, total RNA was reverse transcribed using SuperScript IV reverse transcriptase (Invitrogen). SYBR Green dye (Bio-Rad) was used for qPCR in a Bio-Rad CFX real-time PCR system. The relative mRNA level was determined by normalization with β-actin expression using the 2^-ΔΔCt^ method. Primers used in this study are listed in Supplementary Table 1.

**Subcellular fractionation**

Tissue subcellular fractionation was performed by previously published methods (54,55). Briefly, freshly isolated tissues were washed with cold PBS, transferred into STM buffer (250 mM sucrose, 50 mM Tris-HCl, 5 mM MgCl_2_, and protease/phosphatase inhibitor cocktails), and homogenized using a tissue homogenizer (Omni International). After centrifugation at 800g for 15 min, the pellet was kept for the nuclear fraction and the supernatant was used separating into the mitochondrial and cytosolic/microsomal fractions.

For the nuclear fraction, the pellet was washed two times in STM buffer and resuspended in NET buffer (20 mM HEPES, 1.5 mM MgCl_2_, 0.5 M NaCl, 0.2 mM EDTA, 20% Glycerol, 1% Triton X-100, and protease/phosphatase inhibitor cocktails). After incubation for 30 min on ice, the suspension was passed through the 19-gauge needle, sonicated twice at 20% amplitude for 20 sec, and centrifuged at 9,000g for 30 min. The supernatant was stored at -80°C until use.

For the mitochondrial and cytosolic/microsomal fractions, the supernatant from the first centrifugation (800g) was centrifuged again at 11,000g for 10 min. The resultant pellet was washed once in the STM buffer, resuspended in SOL buffer (50 mM Tris-HCl, 1 mM EDTA, 0.5% Triton X-100, and protease/phosphatase inhibitor cocktails), sonicated three times at 20% amplitude for 10 sec and stored as the mitochondrial fraction. For the cytosolic/microsomal fraction, the supernatant from the second centrifugation (11,000g) was mixed with cold 100% acetone in a 1:1 volume ratio, incubated at -20°C for 1 h, and centrifuged at 12,000g for 5 min. The final pellet was resuspended in STM buffer and kept at -80°C until use. All chemicals were purchased from Sigma.

**Protein extraction and immunoblotting**

For the tissue blot analysis, mouse tissues were collected and homogenized in RIPA buffer supplemented with protease/phosphatase inhibitor cocktails. After centrifugation, the supernatant was collected and quantitated by the BCA method (Pierce BCA protein assay kit, Life Technologies). For western analysis of cultured adipocytes after CL316,243 stimulation, fully differentiated brown adipocytes were treated with 10 µM CL316,243 for 20 min, washed with cold PBS three times, and immediately lysed in RIPA buffer.

Protein samples were loaded on 3­8% or 4-12% SDS-PAGE gels and transferred to nitrocellulose membranes (Bio-Rad) and probed with a primary antibody. We used three CFTR monoclonal antibodies (Cystic Fibrosis Foundation antibody distribution program #450 and #596, 1:1000; CFTR Folding Consortium 3G11; 1:2000), UCP1 monoclonal antibody (R&D systems, 1:3000), FABP4 monoclonal antibody (Santa Cruz, 1:3000), LMNA monoclonal antibody (Santa Cruz, 1:1000), COX IV polyclonal antibody (Sigma, 1:2000), GAPDH monoclonal antibody (Santa Cruz, 1:1000), HSL polyclonal antibody (Cell Signaling Technology, 1:2000), Phospho-HSL (Ser563) polyclonal antibody (Cell Signaling Technology, 1:1000), Cre recombinase antibody (Sigma, 1:1000), β-actin (Santa Cruz, 1:1000), Goat anti-mouse IgG (Jackson ImmunoResearch, 1:5000), and Goat anti-rabbit IgG (Jackson ImmunoResearch, 1:5000). Luminata Crescendo Western HRP substrate (Millipore Sigma) or SuperSignal West Femto Maximum Sensitivity Substrate kit (Fisher Scientific) was used for developing the blots.

**Oxygen consumption rate (OCR) measurement**

The OCR in freshly isolated mature adipocytes and fully differentiated adipocytes were measured using a respirometer (Strathkelvin Instruments, MT200) equipped with a Clark-type electrode (Strathkelvin Instruments, SI130). Cells were incubated in DMEM with 2% fatty acid-free bovine serum albumin (BSA) in the presence of CL316,243 (10 µM). Total cellular protein amounts were determined by the BCA method and used for comparing samples. Data were analyzed by Strathkelvin 782 system data analysis module (version 4.1).

**Glucose tolerance test and insulin tolerance test**

To measure glucose tolerance, mice fed with a high-fat diet (HFD) for 13 weeks were fasted overnight and injected intraperitoneally with glucose at 1 g/kg body weight. For the insulin tolerance test, mice were fasted for 6 h and received intraperitoneal insulin at 0.75 U/kg body weight.

**Lipolysis assay**

Differentiated BAT cells (day 7) derived from CFTR^BATKO^ and WT were trypsinized and suspended in a buffer containing 120 mM NaCl, 5 mM KCl, 1 mM MgCl_2_, 1 mM CaCl_2_, 0.4 mM K_2_HPO_4_, 10 mM glucose, 15 mM NaHCO_3_, 20 mM HEPES, and 4% fatty acid-free BSA. Cells were treated with 10 µM CL for 20 min and the cell suspension was filtered using a 0.45 μm Ultrafree-MC centrifugal filter (Millipore). After centrifugation, the flow-through was used for lipolysis assay using Free Glycerol Reagent (Sigma).

**Measurement of cellular cAMP levels**

Differentiated brown adipocytes in 6-well plates were treated with 10 µM CL316,243 and 0.5 mM IBMX for 5 min at 37°C. Culture media were removed and 1 mL of 0.1M HCl was added to each well to lyse the cells. The cell suspension was loaded to a centrifugal filter unit and centrifuged. The cAMP level was measured using a cyclic AMP ELISA kit (Cayman Chemical).

**Immunohistochemistry**

BAT from UCP1-Cre mice and their littermates were fixed in 10% neutral buffered formalin (Fisher Scientific) overnight, dehydrated, and embedded in paraffin. The paraffin sections were incubated in citric acid at 95^o^C for 18 min for antigen retrieval. The sections were incubated with Cre recombinase antibody (Sigma, 1:1000) overnight at 4^o^C, then with biotinylated mouse secondary antibody (1:1000) for 1 hour at RT, and subsequently developed for 2 min using DAB solution (Vector Laboratories).

**Supplementary Table 1. Primers used in this study**

| **Primer** | **Forward (5' to 3')** | **Reverse (5' to 3')** | **Purpose** |
| --- | --- | --- | --- |
| Cftr_WT | GTAGGGGCTCGCTCTTCTTT | GTACCCGGCATAATCCAAGA | Genotyping |
| Cftr_KO | GTAGGGGCTCGCTCTTCTTT | AGCCCCTCGAGGGACCTAAT | Genotyping |
| Cftr | TGAGGAGGACAGGGATGATAA | GAACCACACAGATGGGTACAA | qPCR |
| Ucp1 | ACTGCCACACCTCCAGTCATT | CTTTGCCTCACTCAGGATTGG | qPCR |
| Cidea | TGCTCTTCTGTATCGCCCAGT | GCCGTGTTAAGGAATCTGCTG | qPCR |
| Cox5a | GGGTCACACGAGACAGATGA | GGAACCAGATCATAGCCAACA | qPCR |
| Pgc1α | GAAAGGGCCAAACAGAGAGA | GTAAATCACACGGCGCTCTT | qPCR |
| Prdm16 | CAGCACGGTGAAGCCATTC | GCGTGCATCCGCTTGTG | qPCR |
| Atgl | CAGCACATTTATCCCGGTGTAC | AAATGCCGCCATCCACATAG | qPCR |
| Hsl | GCTGGGCTGTCAAGCACTGT | GTAACTGGGTAGGCTGCCAT | qPCR |
| β-Actin | CTAAGGCCAACCGTGAAAAG | ACCAGAGGCATACAGGGACA | qPCR (normalization control) |

**Supplementary Figure 1. DNA analysis from different adipose depots in CFTR^BATKO^ mice and WT littermates.**

The PCR amplicon at 408 bp indicates the floxed CFTR allele and the amplicon at 154 bp marks the excised exon 11 allele. In adipose tissues, non-adipocytes such as endothelial cells, immune cells, stem cells, and neurons make up about half of the cell population (56). Accordingly, BAT shows both the floxed CFTR allele from non-adipocytes and the excised exon 11 allele from adipocytes. Inguinal WAT (iWAT) and epididymal WAT (eWAT) only show the floxed CFTR allele.

**Supplementary Figure 2. Loss of CFTR protein in BAT from CFTR^BATKO^ mice.**

(A) Immunohistochemical analysis of Cre recombinase expression in BAT from CFTR^BATKO^ mice and their WT littermates. Cre expression is seen in most cells in KO BAT but is absent in WT BAT.

(B) Western analysis of CFTR expression in BAT from CFTR^BATKO^ mouse and WT littermate. 3G11 CFTR antibody targeting the NBD1 region was used.


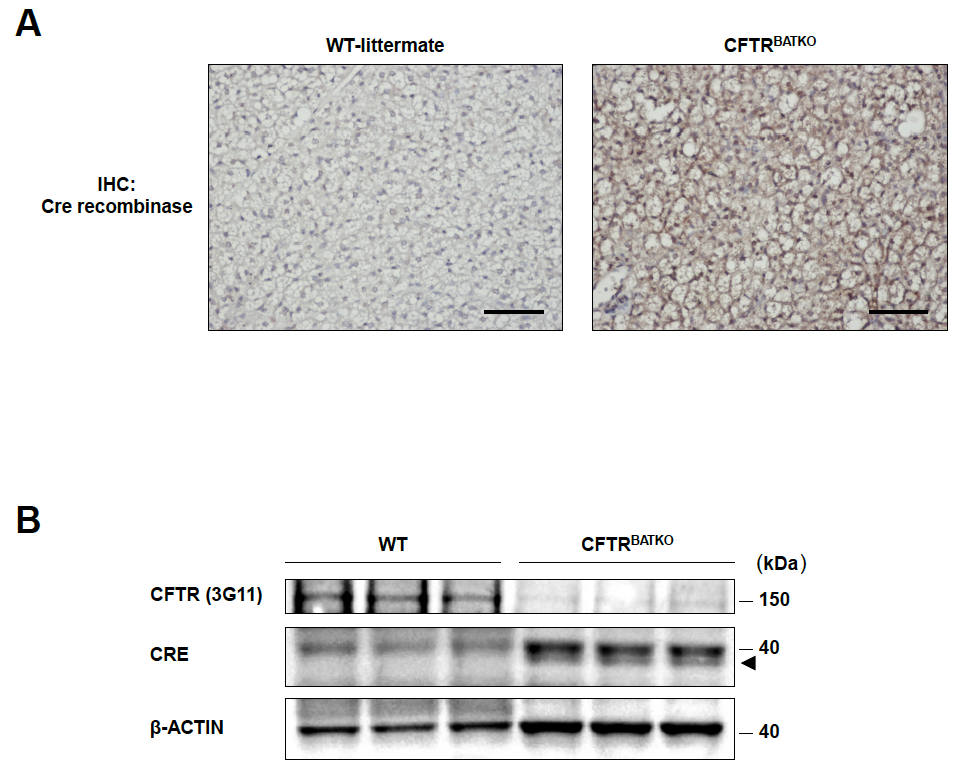


**Supplementary Figure 3. Metabolic studies in the CFTR^BATKO^ mice.**

(A) Oxygen consumption in CFTR^BATKO^ mice and WT littermates at three different ambient temperatures of 30^o^C, 22^o^C, and 10^o^C. n = 9 per group.

(B) Carbon dioxide production. n = 9 per group.

(C) Respiratory exchange ratio. n = 9 per group.

(D) Daily food intake. n = 9 per group.

(E) Physical activity. n = 9 per group.

(F) Calculated carbohydrate oxidation rates. n = 9 per group.

(G) Calculated fat oxidation rates. n = 9 per group.

(H) Daily food intake by CFTR^BATKO^ and WT mice during a HFD study. WT, n = 9; CFTR^BATKO^, n = 10.

Data are presented as mean ± s.e.m. *P*-values are determined by two-tailed Student’s t-test (C-H) or two-way ANOVA with post-hoc Tukey test (A, B). *p < 0.05; **p < 0.01; ***p < 0.001.


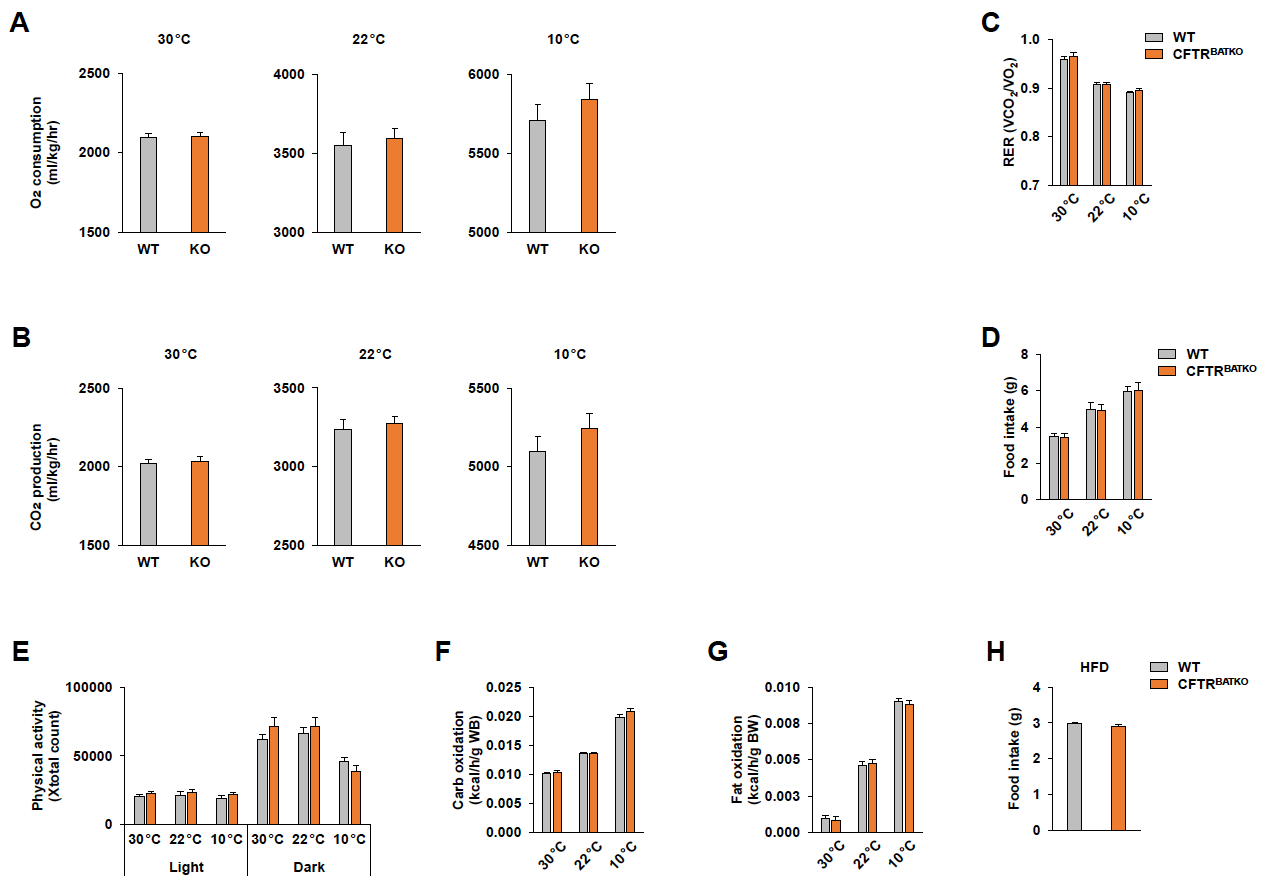


**Supplementary Figure 4. UCP1-Cre transgene expression does not alter systemic energy expenditure in mice.**

(A) Body composition analysis in 12-week-old Ucp1-Cre mice and their WT littermate controls fed regular chow at 22^o^C. n = 7 per group.

(B) Average energy expenditure (Kcal/h) of 12-week-old Ucp1-Cre mice and WT littermate controls at 22^o^C and 10^o^C environmental temperatures. n = 7 per group.

(C) Respiratory exchange ratio. n = 7 per group.

(D) Food intake. n = 7 per group.

(E) Physical activity. n = 7 per group.

(F) Core rectal temperature of singly housed 10-week-old mice upon transition from 22^o^C to 10^o^C. WT, n= 4; Ucp1-Cre, n = 4.

Data are presented as mean ± s.e.m. *P*-values are determined by two-tailed Student’s t-test (A,C-F) or two-way ANOVA with post-hoc Tukey test (B). *p < 0.05; **p < 0.01; ***p < 0.001.


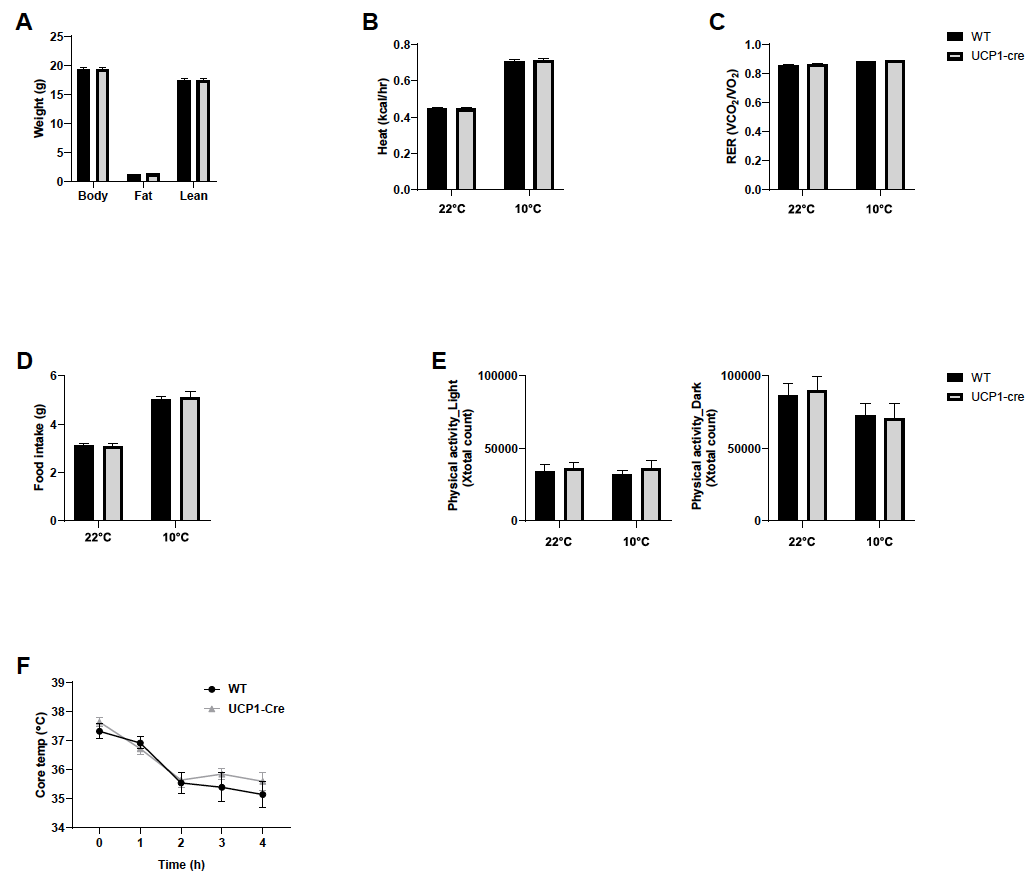


**Supplementary References**

54. Cox B, Emili A. Tissue subcellular fractionation and protein extraction for use in mass-spectrometry-based proteomics. Nat Protoc. 2006; 1(4):1872–8.

55. Dimauro I, Pearson T, Caporossi D, Jackson MJ. A simple protocol for the subcellular fractionation of skeletal muscle cells and tissue. BMC Res Notes. 2012; 5:513.

56. Roh HC, Tsai LTY, Lyubetskaya A, Tenen D, Kumari M, Rosen ED. Simultaneous transcriptional and epigenomic profiling from specific cell types within heterogeneous tissues in vivo. Cell Rep 2017; 18(4):1048-1061.
